# Diaqua­bis­(pyridine-2-sulfonato-κ^2^ *N*,*O*)cobalt(II)

**DOI:** 10.1107/S1600536811048203

**Published:** 2011-11-19

**Authors:** Zong-Sheng Li, Seik Weng Ng

**Affiliations:** a College of Safety and Environment Engineering, Capital University of Economics and Business, Beijing 100070, People’s Republic of China; b Department of Chemistry, University of Malaya, 50603 Kuala Lumpur, Malaysia; c Chemistry Department, Faculty of Science, King Abdulaziz University, PO Box 80203 Jeddah, Saudi Arabia

## Abstract

The title complex, [Co(C_5_H_4_NO_3_S)_2_(H_2_O)_2_], lies on a twofold rotation axis that relates the two water mol­ecules and the two pyridine-2-sulfonate ions. The Co^II^ atom exists in an slightly distorted octa­hedral environment. The N-donor atoms are *cis* to each other. In the crystal, adjacent mol­ecules are linked by O—H⋯O hydrogen bonds into a layer motif extending along (001).

## Related literature

For the isotypic manganese(II), zinc and cadmium analogs, see: Lobana *et al.* (2004 ▶); Xiao (2007 ▶); Xiao & Liu (2004 ▶).
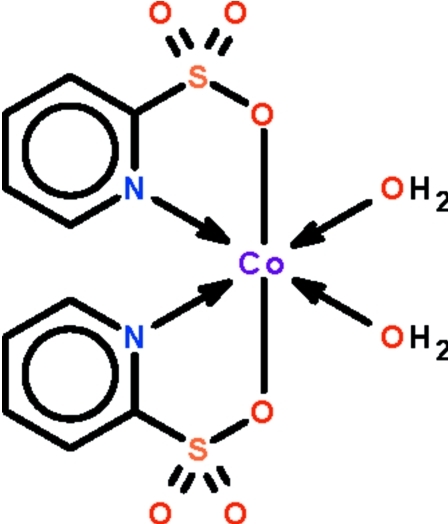


## Experimental

### 

#### Crystal data

[Co(C_5_H_4_NO_3_S)_2_(H_2_O)_2_]
*M* *_r_* = 411.29
Monoclinic, 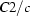

*a* = 13.7009 (9) Å
*b* = 7.1127 (5) Å
*c* = 16.0180 (11) Å
β = 106.734 (1)°
*V* = 1494.86 (18) Å^3^
*Z* = 4
Mo *K*α radiation
μ = 1.47 mm^−1^
*T* = 296 K
0.25 × 0.20 × 0.15 mm

#### Data collection

Bruker SMART APEX diffractometer
Absorption correction: multi-scan (*SADABS*; Sheldrick, 1996 ▶) *T*_min_ = 0.710, *T*_max_ = 0.810
4331 measured reflections
1695 independent reflections
1590 reflections with *I* > 2σ(*I*)
*R*_int_ = 0.016

#### Refinement

*R*[*F* ^2^ > 2σ(*F* ^2^)] = 0.024
*wR*(*F* ^2^) = 0.067
*S* = 1.03
1695 reflections
113 parameters
3 restraints
H atoms treated by a mixture of independent and constrained refinement
Δρ_max_ = 0.35 e Å^−3^
Δρ_min_ = −0.34 e Å^−3^

### 

Data collection: *APEX2* (Bruker, 2007 ▶); cell refinement: *SAINT* (Bruker, 2007 ▶); data reduction: *SAINT*; program(s) used to solve structure: *SHELXS97* (Sheldrick, 2008 ▶); program(s) used to refine structure: *SHELXL97* (Sheldrick, 2008 ▶); molecular graphics: *X-SEED* (Barbour, 2001 ▶); software used to prepare material for publication: *publCIF* (Westrip, 2010 ▶).

## Supplementary Material

Crystal structure: contains datablock(s) global, I. DOI: 10.1107/S1600536811048203/zs2164sup1.cif

Structure factors: contains datablock(s) I. DOI: 10.1107/S1600536811048203/zs2164Isup2.hkl

Additional supplementary materials:  crystallographic information; 3D view; checkCIF report

## Figures and Tables

**Table 1:** Hydrogen-bond geometry (Å, °)

*D*—H⋯*A*	*D*—H	H⋯*A*	*D*⋯*A*	*D*—H⋯*A*
O1w—H11⋯O2^i^	0.83 (1)	1.90 (1)	2.735 (2)	177 (3)
O1w—H12⋯O3^ii^	0.83 (1)	1.88 (1)	2.703 (2)	172 (3)

Symmetry codes: (i) 
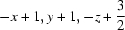
; (ii) 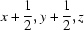.

**Fig. 1.**
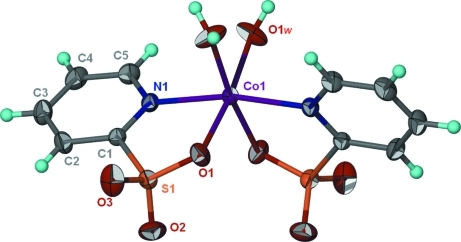
Thermal ellipsoid plot (Barbour, 2001) of [Co(H2O)2(C5H4NO3S)2] at the 50% probability level. Hydrogen atoms are drawn as spheres of arbitrary radius. The unlabeled atoms are related to the labeled ones by twofold rotational symmetry (symmetry code -x + 1, y, -z + 3/2).

**Fig. 2.**
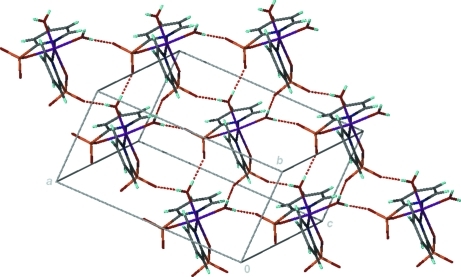
The hydrogen-bonded layer structure.

## supplementary crystallographic information

### Comment

Diaquabis(pyridine-2-sulfonate)cobalt(II) (Scheme I) is isostructural with the manganese, zinc (Lobana *et al.*, 2004; Xiao & Liu, 2004) and cadmium (Xiao, 2007) analogs. The molecule lies on a twofold rotation axis that relates the two water molecules and the two pyridine-2-sulfonate ions and the Co^II^ atom exist in an slightly distorted octahedral environment. The N donor atoms are *cis* to each other (Fig. 1). Adjacent molecules are linked by water O–H···O_sulfonate_ hydrogen bonds (Table 1) into a layer motif extending along (0 0 1) (Fig. 2).

### Experimental

Pyridine-2-sulfonic acid (0.4 mmol, 0.0641 g) was dissolved in 0.1 *M* sodium hydroxide (4 ml); cobalt(II) chloride hexahydrate (0.2 mmol, 0.0476 g) and 4,4'-bipyridine-*N*,*N*'-dioxide (0.2 mmol, 0.0446 g) were added to the solution. The clear solution was allowed to evaporate at ambient conditions, affording red block-shaped crystals after one week, in 40% yield.

### Refinement

Carbon-bound H-atoms were placed in calculated positions (C–H 0.93 Å) and were included in the refinement in the riding model approximation, with *U*_iso_(H) set to 1.2*U*_eq_(C). The water H-atoms were located in a difference Fourier map and was refined with distance restraints of O—H = 0.83±0.01 and H···H = 1.37±0.01 Å. Their isotropic displacement parameters were refined.

### Crystal data

**Table:**

[Co(C_5_H_4_NO_3_S)_2_(H_2_O)_2_]	*F*(000) = 836
*M**_r_* = 411.29	*D*_x_ = 1.827 Mg m^−^^3^
Monoclinic, *C*2/*c*	Mo *K*α radiation, λ = 0.71073 Å
Hall symbol: -C 2yc	Cell parameters from 3169 reflections
*a* = 13.7009 (9) Å	θ = 3.1–27.6°
*b* = 7.1127 (5) Å	µ = 1.47 mm^−^^1^
*c* = 16.0180 (11) Å	*T* = 296 K
β = 106.734 (1)°	Prism, yellow
*V* = 1494.86 (18) Å^3^	0.25 × 0.20 × 0.15 mm
*Z* = 4	

### Data collection

**Table:**

Bruker SMART APEX diffractometer	1695 independent reflections
Radiation source: fine-focus sealed tube	1590 reflections with *I* > 2σ(*I*)
graphite	*R*_int_ = 0.016
ω scans	θ_max_ = 27.5°, θ_min_ = 2.7°
Absorption correction: multi-scan (*SADABS*; Sheldrick, 1996)	*h* = −17→11
*T*_min_ = 0.710, *T*_max_ = 0.810	*k* = −9→9
4331 measured reflections	*l* = −13→20

### Refinement

**Table:**

Refinement on *F*^2^	Primary atom site location: structure-invariant direct methods
Least-squares matrix: full	Secondary atom site location: difference Fourier map
*R*[*F*^2^ > 2σ(*F*^2^)] = 0.024	Hydrogen site location: inferred from neighbouring sites
*wR*(*F*^2^) = 0.067	H atoms treated by a mixture of independent and constrained refinement
*S* = 1.03	*w* = 1/[σ^2^(*F*_o_^2^) + (0.0362*P*)^2^ + 1.5779*P*] where *P* = (*F*_o_^2^ + 2*F*_c_^2^)/3
1695 reflections	(Δ/σ)_max_ = 0.001
113 parameters	Δρ_max_ = 0.35 e Å^−^^3^
3 restraints	Δρ_min_ = −0.34 e Å^−^^3^

### Fractional atomic coordinates and isotropic or equivalent isotropic displacement parameters (Å^2^)

**Table:**

	*x*	*y*	*z*	*U*_iso_*/*U*_eq_	
Co1	0.5000	0.69090 (4)	0.7500	0.02354 (11)	
S1	0.33529 (3)	0.39913 (6)	0.66183 (2)	0.02716 (12)	
O1	0.38590 (11)	0.4900 (2)	0.74481 (7)	0.0379 (3)	
O2	0.37681 (13)	0.21672 (19)	0.65256 (10)	0.0474 (4)	
O3	0.22560 (11)	0.3995 (3)	0.64164 (10)	0.0504 (4)	
O1W	0.59993 (12)	0.9037 (2)	0.74438 (11)	0.0467 (4)	
H11	0.6059 (18)	1.001 (2)	0.7743 (14)	0.055 (7)*	
H12	0.6431 (16)	0.902 (3)	0.7169 (15)	0.060 (8)*	
N1	0.44352 (10)	0.6597 (2)	0.61055 (9)	0.0255 (3)	
C1	0.36597 (12)	0.5407 (2)	0.58114 (9)	0.0237 (3)	
C2	0.31435 (14)	0.5162 (3)	0.49417 (11)	0.0339 (4)	
H2	0.2589	0.4352	0.4769	0.041*	
C3	0.34772 (16)	0.6160 (3)	0.43366 (11)	0.0398 (4)	
H3	0.3149	0.6034	0.3744	0.048*	
C4	0.43020 (15)	0.7344 (3)	0.46221 (12)	0.0384 (4)	
H4	0.4551	0.7998	0.4224	0.046*	
C5	0.47534 (14)	0.7548 (3)	0.55036 (12)	0.0344 (4)	
H5	0.5299	0.8374	0.5691	0.041*	

### Atomic displacement parameters (Å^2^)

**Table:**

	*U*^11^	*U*^22^	*U*^33^	*U*^12^	*U*^13^	*U*^23^
Co1	0.02474 (17)	0.02440 (17)	0.02083 (16)	0.000	0.00555 (11)	0.000
S1	0.0294 (2)	0.0293 (2)	0.0240 (2)	−0.00801 (15)	0.00954 (16)	0.00137 (14)
O1	0.0506 (8)	0.0432 (7)	0.0214 (5)	−0.0189 (6)	0.0125 (5)	−0.0010 (5)
O2	0.0711 (11)	0.0282 (7)	0.0465 (8)	0.0001 (7)	0.0228 (7)	0.0063 (6)
O3	0.0306 (7)	0.0803 (12)	0.0433 (8)	−0.0137 (7)	0.0154 (6)	0.0049 (7)
O1W	0.0496 (9)	0.0409 (8)	0.0625 (9)	−0.0211 (7)	0.0367 (8)	−0.0254 (7)
N1	0.0268 (7)	0.0279 (7)	0.0218 (6)	−0.0028 (5)	0.0068 (5)	0.0036 (5)
C1	0.0262 (7)	0.0245 (7)	0.0216 (7)	0.0003 (6)	0.0088 (6)	0.0013 (6)
C2	0.0371 (9)	0.0382 (9)	0.0246 (8)	−0.0068 (8)	0.0060 (7)	−0.0044 (7)
C3	0.0489 (11)	0.0493 (11)	0.0201 (7)	0.0006 (9)	0.0081 (7)	0.0008 (7)
C4	0.0488 (11)	0.0418 (10)	0.0291 (8)	0.0012 (9)	0.0184 (8)	0.0109 (8)
C5	0.0364 (9)	0.0346 (9)	0.0335 (9)	−0.0077 (8)	0.0119 (7)	0.0070 (7)

### Geometric parameters (Å, °)

**Table:**

Co1—O1W	2.0601 (14)	O1W—H12	0.832 (9)
Co1—O1W^i^	2.0601 (13)	N1—C1	1.334 (2)
Co1—O1	2.1018 (13)	N1—C5	1.349 (2)
Co1—O1^i^	2.1018 (13)	C1—C2	1.380 (2)
Co1—N1	2.1545 (13)	C2—C3	1.381 (3)
Co1—N1^i^	2.1545 (13)	C2—H2	0.9300
S1—O2	1.4414 (15)	C3—C4	1.378 (3)
S1—O3	1.4432 (14)	C3—H3	0.9300
S1—O1	1.4612 (13)	C4—C5	1.376 (3)
S1—C1	1.7814 (15)	C4—H4	0.9300
O1W—H11	0.833 (9)	C5—H5	0.9300
			
O1W—Co1—O1W^i^	85.45 (9)	Co1—O1W—H11	122.9 (16)
O1W—Co1—O1	173.62 (6)	Co1—O1W—H12	126.6 (16)
O1W^i^—Co1—O1	90.29 (6)	H11—O1W—H12	110.3 (15)
O1W—Co1—O1^i^	90.29 (6)	C1—N1—C5	117.02 (14)
O1W^i^—Co1—O1^i^	173.61 (6)	C1—N1—Co1	116.31 (10)
O1—Co1—O1^i^	94.34 (8)	C5—N1—Co1	126.58 (12)
O1W—Co1—N1	94.31 (6)	N1—C1—C2	124.14 (15)
O1W^i^—Co1—N1	94.36 (6)	N1—C1—S1	115.65 (11)
O1—Co1—N1	81.26 (5)	C2—C1—S1	120.12 (13)
O1^i^—Co1—N1	90.69 (5)	C1—C2—C3	117.83 (17)
O1W—Co1—N1^i^	94.36 (6)	C1—C2—H2	121.1
O1W^i^—Co1—N1^i^	94.31 (6)	C3—C2—H2	121.1
O1—Co1—N1^i^	90.69 (5)	C4—C3—C2	119.14 (16)
O1^i^—Co1—N1^i^	81.26 (5)	C4—C3—H3	120.4
N1—Co1—N1^i^	168.19 (8)	C2—C3—H3	120.4
O2—S1—O3	113.26 (10)	C3—C4—C5	119.23 (17)
O2—S1—O1	113.20 (9)	C3—C4—H4	120.4
O3—S1—O1	113.19 (9)	C5—C4—H4	120.4
O2—S1—C1	104.56 (8)	N1—C5—C4	122.56 (17)
O3—S1—C1	106.52 (8)	N1—C5—H5	118.7
O1—S1—C1	105.11 (7)	C4—C5—H5	118.7
S1—O1—Co1	119.31 (7)		
			
O2—S1—O1—Co1	98.03 (11)	C5—N1—C1—C2	−3.1 (3)
O3—S1—O1—Co1	−131.35 (10)	Co1—N1—C1—C2	173.84 (14)
C1—S1—O1—Co1	−15.48 (11)	C5—N1—C1—S1	173.39 (13)
O1W^i^—Co1—O1—S1	104.28 (10)	Co1—N1—C1—S1	−9.71 (16)
O1^i^—Co1—O1—S1	−80.13 (9)	O2—S1—C1—N1	−103.32 (14)
N1—Co1—O1—S1	9.91 (10)	O3—S1—C1—N1	136.50 (14)
N1^i^—Co1—O1—S1	−161.41 (10)	O1—S1—C1—N1	16.13 (15)
O1W—Co1—N1—C1	−174.23 (12)	O2—S1—C1—C2	73.28 (16)
O1W^i^—Co1—N1—C1	−88.48 (12)	O3—S1—C1—C2	−46.89 (17)
O1—Co1—N1—C1	1.14 (12)	O1—S1—C1—C2	−167.27 (15)
O1^i^—Co1—N1—C1	95.43 (12)	N1—C1—C2—C3	2.6 (3)
N1^i^—Co1—N1—C1	48.64 (11)	S1—C1—C2—C3	−173.66 (14)
O1W—Co1—N1—C5	2.33 (15)	C1—C2—C3—C4	0.0 (3)
O1W^i^—Co1—N1—C5	88.09 (15)	C2—C3—C4—C5	−2.0 (3)
O1—Co1—N1—C5	177.71 (16)	C1—N1—C5—C4	0.9 (3)
O1^i^—Co1—N1—C5	−88.01 (15)	Co1—N1—C5—C4	−175.65 (14)
N1^i^—Co1—N1—C5	−134.79 (15)	C3—C4—C5—N1	1.6 (3)

Symmetry codes: (i) −*x*+1, *y*, −*z*+3/2.

### Hydrogen-bond geometry (Å, °)

**Table:**

*D*—H···*A*	*D*—H	H···*A*	*D*···*A*	*D*—H···*A*
O1w—H11···O2^ii^	0.83 (1)	1.90 (1)	2.735 (2)	177 (3)
O1w—H12···O3^iii^	0.83 (1)	1.88 (1)	2.703 (2)	172 (3)

Symmetry codes: (ii) −*x*+1, *y*+1, −*z*+3/2; (iii) *x*+1/2, *y*+1/2, *z*.
